# Use of national standards to monitor HIV care and treatment in a high prevalence city—Washington, DC

**DOI:** 10.1371/journal.pone.0186036

**Published:** 2017-10-05

**Authors:** Amanda D. Castel, Arpi Terzian, Rachel Hart, Nabil Rayeed, Mariah M. Kalmin, Heather Young, Alan E. Greenberg, for the DC Cohort Executive Committee

**Affiliations:** 1 Department of Epidemiology and Biostatistics, Milken Institute School of Public Health, George Washington University, Washington, DC., United States of America; 2 Cerner Corporation, Kansas City, Missouri, United States of America; ARGENTINA

## Abstract

We sought to benchmark the quality of HIV care being received by persons living with HIV in care in Washington, DC and identify individual-level and structural-level differences. Data from the DC Cohort, an observational HIV cohort of persons receiving outpatient care in DC, were used to estimate the Institute of Medicine (IOM) and Department of Health and Human Services (HHS) quality of care measures. Differences in care by demographics and clinic type were assessed using χ2 tests and multivariable regression models. Among 8,047 participants, by HHS standards, 69% of participants were retained in care (RIC), 95% were prescribed antiretroviral therapy (ART), and 84% were virally suppressed (VS). By IOM standards, 84% were in continuous care; and 78% and 80% underwent regular CD4 and VL monitoring, respectively. Screening for syphilis, chlamydia, and gonorrhea was 51%, 31%, and 26%, respectively. Older participants were 1.5 times more likely to be RIC compared to younger participants (OR: 1.5; 95% CI: 1.3, 1.8). Participants enrolled in community-based clinics were more likely to be RIC (OR: 1.7; 95% CI: 1.4, 2.0) versus those enrolled at hospital-based clinics. Older participants were more likely to achieve VS than younger participants (OR: 1.8; 95% CI: 1.5, 2.2) while Black participants were less likely compared to white participants (OR: 0.4; 95% CI: 0.3, 0.5). Despite high measures of quality of care, disparities remain. Continued monitoring of the quality of HIV care and treatment can inform the development of public health programs and interventions to optimize care delivery.

## Introduction

The US National HIV/AIDS Strategy has established specific targets for persons living with HIV/AIDS (PLWHA) which include having 90% of people living with HIV diagnosed, 90% retained in HIV medical care, 80% achieving viral suppression, and ensuring 95% of PLWHA have stable housing [1]. In order to measure progress in achieving those targets, established HIV clinical cohorts and federally-funded HIV/AIDS programs are able to measure the quality of HIV clinical care along the HIV care continuum, a widely-adopted public health framework summarizing sequential steps from HIV diagnosis to optimal care resulting in viral suppression [2–4]. Clinical cohorts such as the North American AIDS Cohort Collaboration (NA-ACCORD), as well as local and national HIV surveillance registries, use a set of core indicators including those developed by the Institute of Medicine (IOM) and the Department of Health and Human Services (HHS), to track and compare the delivery of care within and across other, similar populations of PLWHA [5–8].

In Washington, DC- a city with a 2.5% HIV prevalence- the measurement and assessment of care delivery at a population-level is facilitated by a unique study, the DC Cohort study. The DC Cohort is a longitudinal, observational study of PLWHA receiving HIV care at 13 of the large publicly-funded, government, and academic medical center clinics in Washington, DC [9]. The DC Cohort, which provides representative data on PLWHA persons living with HIV in DC, follows participants receiving outpatient HIV/AIDS care by combining their clinical data into a centralized database. Given the study’s unique ability to aggregate data and capture care delivery at a diverse set of HIV clinics in a highly impacted area, the Cohort has been identified as a comprehensive data source to monitor HIV care in DC [10].

Consistent with the overarching goals of the DC Cohort and in support of the Affordable Care Act (ACA) that prioritizes the delivery and tracking of high-quality patient care, the objectives of this study were to: 1) benchmark the quality of HIV care being delivered at DC Cohort sites by applying HHS and IOM quality of care indicators for retention in HIV medical care, antiretroviral therapy (ART use), HIV viral load (VL) suppression and other measures; and 2) determine whether individual-level predictors such as age, sex, race/ethnicity, HIV transmission risk and structural–level predictors such as clinic type influence quality of care measures [11, 12].

## Methods

### Study population

The DC Cohort began enrollment in January 2011; 8,047 consenting PLWHA who receive HIV care at one or more participating clinics, independent of DC residence status, were enrolled as of September 30, 2016. Of the 13 DC Cohort sites, eight are hospital-based and five are community-based clinics. Participants’ clinical data were abstracted from patient medical records and entered into a web-based data entry system called Discovere® (Cerner Corporation, Kansas City, MO). The study protocol, consent forms, and research instruments were approved by the George Washington University Institutional Review Board (IRB), the DC Department of Health (DOH) IRB, and the IRBs of Howard University, Georgetown University, Medstar Research Institute, and the Veterans Affairs Medical Center. Reliance agreements with the GWU IRB were established with the remaining clinical sites. Details of the DC Cohort study design have been described previously [10, 12, 13].

### Outcomes

Among the HHS and IOM indicators, four out of seven HHS-defined indicators and seven out of nine IOM-defined indicators were selected based on what could feasibly be measured using existing data collected in the DC Cohort. Data were collected on participants enrolled as of September 30, 2016 and reported to the DC Cohort as of December 15, 2016. Measures were computed among those who had ≥12 months of follow-up, with the exception of the HHS indicator on retention-in-care which required ≥24 months of follow-up [2, 4]. For measures requiring care visits within a specified interval of time, in addition to documented clinical encounters, CD4 or VL test results served as proxies for HIV primary care encounters, a standard practice used by HIV cohorts and surveillance programs [14–17].

In 2012, HHS developed a set of common, core measures to estimate outcomes along the HIV care continuum as part of a larger initiative to strengthen the federal response to HIV/AIDS through planning and evaluating domestic HIV/AIDS programs within and across agencies. Based on an assessment of federally-funded HIV prevention, treatment, and care services, HHS identified core indicators related to continuous HIV clinical care and access to supportive services. Selected core indicators were measured, including retention in care, ART use, viral load suppression, and housing status (Table 1) [2]. Similarly, the IOM has also developed a set of core indicators related to continuous HIV clinical care as part of an initiative to monitor the effect of both the National HIV/AIDS Policy and ACA on improving HIV care [1, 11]. Select core indicators were measured, including continuous care, regular CD4 and viral monitoring, maintenance of immune function, and screening for chlamydia, gonorrhea, and syphilis (Table 1) [4].

**Table 1:** HHS and IOM quality of care indicators assessed using DC Cohort study data^a^.

HHS Measure	HHS definition	IOM measure	IOM definition
**Retention-in-care**	Proportion of HIV+ persons who had ≥1 HIV medical care visit in each 6-month period of the 24 month measurement period, with ≥60 days between the first medical visit in the prior 6 month period and the last medical visit in the subsequent 6-month period among those with ≥24 months of follow-up	**Proportion in continuous HIV care**	Proportion of HIV+ people who had ≥2 routine HIV medical care visits or a CD4 or viral load test in the preceding 12 months ≥ 3 months apart among those with ≥12 months follow-up
**ARV therapy among persons in HIV medical care**	Proportion of HIV+ persons who are prescribed ART in the 12-month measurement period among those with ≥12 months follow-up and ≥1 visit	**Regular CD4 testing for monitoring immune function**	Proportion of HIV+ people who received ≥2 CD4 tests in 12 months since enrollment among those with ≥12 months follow-up
**Viral load (VL) suppression among persons in care**	Proportion of HIV+ persons with a viral load <200 copies/ml at last test in the 12–month measurement period among those with ≥12 months follow-up and ≥ 1 visit and ≥ 1 VL	**Regular VL monitoring for clinical progression**	Proportion of HIV+ people receiving ≥≥2 VL tests in 12 months since enrollment among those with ≥12 months follow-up
**Housing instability**	Proportion of HIV+ persons who were homeless or unstably housed in the 12-month measurement period among those with ≥12 months follow-up with housing information recorded	**Maintenance of immune function to reduce risk of opportunistic infections and cancer**	Proportion of HIV+ people in continuous care for ≥12 months and with a CD4+ cell count ≥350 cells/mm^3^ among those in continuous care for ≥ 12 months
		**Screening for Gonorrhea**	Proportion of HIV+ people who were screened for Gonorrhea ≥1 since enrollment among those with ≥12 months follow-up
		**Screening for Chlamydia**	Proportion of HIV+ people who were screened Chlamydia ≥1 since enrollment among those with ≥12 months follow-up
		**Screening for Syphilis**	Proportion of HIV+ people who were screened for Syphilis ≥1 since enrollment among those with ≥12 months follow-up

^a^IOM = Institute of Medicine; HHS = Department of Health and Human Services.

### Statistical analyses

Frequencies on demographic and clinical characteristics at study enrollment (‘baseline’) were computed. HIV-related clinical characteristics such as history of AIDS, baseline CD4 and VL results (defined as the closest lab result reported within six months prior and 30 days after enrollment), ART exposure status and regimen type (among those prescribed ART) were also measured. HHS and IOM indicators were computed and chi-square test statistics were used to determine differences in six selected indicators by demographic and clinical characteristics. Multivariable logistic regression models were used to estimate adjusted odds ratios (OR) and 95% confidence intervals (CI); models included age, sex at birth, race/ethnicity, HIV transmission risk group and clinic type (hospital versus community-based). Bonferroni correction was applied to account for multiple testing; p-values <0.0083 (i.e., 0.05/6) were considered as the threshold for statistical significance. All analyses were completed using SAS version 9.4 (SAS Institute, Cary, NC).

## Results

### Participant characteristics

Among the 8,047 participants enrolled by September 2016, 91% and 62% had at least 12 and 24 months of follow up, respectively. The proportion of participants who were non-Hispanic black was 78% and the median age was 47 years. Most were publically insured (63%); more than half were receiving HIV care at community-based clinics (53%) and 75% were DC residents (Table 2). The most common mode of HIV transmission was men who had sex with men (MSM; 39%), followed by heterosexual risk (32%).

**Table 2:** Demographic and clinical characteristics at enrollment, DC Cohort Study, Washington, DC 2011–2016^a^^,^^b^.

	Analytic population	
	N	%
**Total**	8,047	100
**Age at enrollment**		
Median (IQR)	47.0 (36.3, 54.6)	
**Sex at birth**		
Female	2,209	27.5
Male	5,838	72.5
**Race/ethnicity**		
Black	6,250	77.7
White	1,155	14.4
Other/Unknown^b^	642	8.0
**Insurance**		
Private	2,076	25.8
Public	5,079	63.1
Other	158	2.0
Unknown	734	9.1
**State of residence**		
DC	5,994	74.5
MD	1,494	18.6
VA	453	5.6
WV	34	0.4
Other/Unknown	72	0.9
**Clinic type**		
Community-based	4,282	53.2
Hospital -based	3,765	46.8
**Housing status**		
Permanent	5,228	65.0
Homeless/Temporary	630	7.8
Other/Unknown	2,189	27.2
**HIV risk**^**c**^		
MSM	3,129	38.9
Heterosexual	2,588	32.2
IDU	541	6.7
Other	396	4.9
Unknown	1,393	17.3
**AIDS diagnosis**	3,403	42.3
**CD4 count (cells/μl)**		
<50	223	2.8
50–199	663	8.2
200–499	2,752	34.2
500**+**	4,182	52.0
Unknown	227	2.8
**VL (copies/ml)**		
<200 200–399 00–9,999 10,000–99,9999 ≥100,000 Unknown	5,870 227 707 675 329 239	72.9 2.8 8.8 8.4 4.1 3.0
**ARV status**		
Prescribed ot prescribed Unknown	7,424 542 81	92.3 6.7 1.0
**ARV regimens among those prescribed (at enrollment)**		
PI-based	2,299	31.0
NNRTI-based	2,107	28.4
Dual class regimens	676	9.1
INSTI-based	966	13.0
NRTI only regimens	166	2.2
Triple class regimens	149	2.0
Other regimens^d^	8	0.1
No recorded ARV regimen at baseline	1,053	14.2

^a^Includes enrolled participants who consented as of September 30, 2016. Data reported to the DC HIV Cohort study as of December 15, 2016. Baseline demographic characteristics reflect data reported at study enrollment.

^b^Other race includes races reported as Hispanic, Asian, American Indian/Native American, Hawaiian/Other Pacific Islander, White-Black, and Other.

^c^MSM risk includes persons identified as having both MSM and injection drug use risk. Other risk includes perinatal transmission (65%), transfusion/coagulation disorder (19%), hemophiliacs and risk due to occupational exposure and other (16%).

^d^Other ARV regimens include TDF+FTC+MVC, 3TC+ABC+TDF+MVC, and AZT+TDF+FTC+MVC

Notes: IQR = interquartile range; NH = non-Hispanic; MSM = men who have sex with men; IDU = male or female injection drug user. VL = viral load; ARV = antiretroviral therapy.

At enrollment, 52% of participants had a CD4 count above 500 cells/μl, and 42% of participants had a history of AIDS. The proportion of treatment prescribed participants was 92% at enrollment (Table 2). Seventy-three percent of participants had a VL at enrollment that was less than 200 copies/ml.

### Indicator measurement

**Fig 1:**
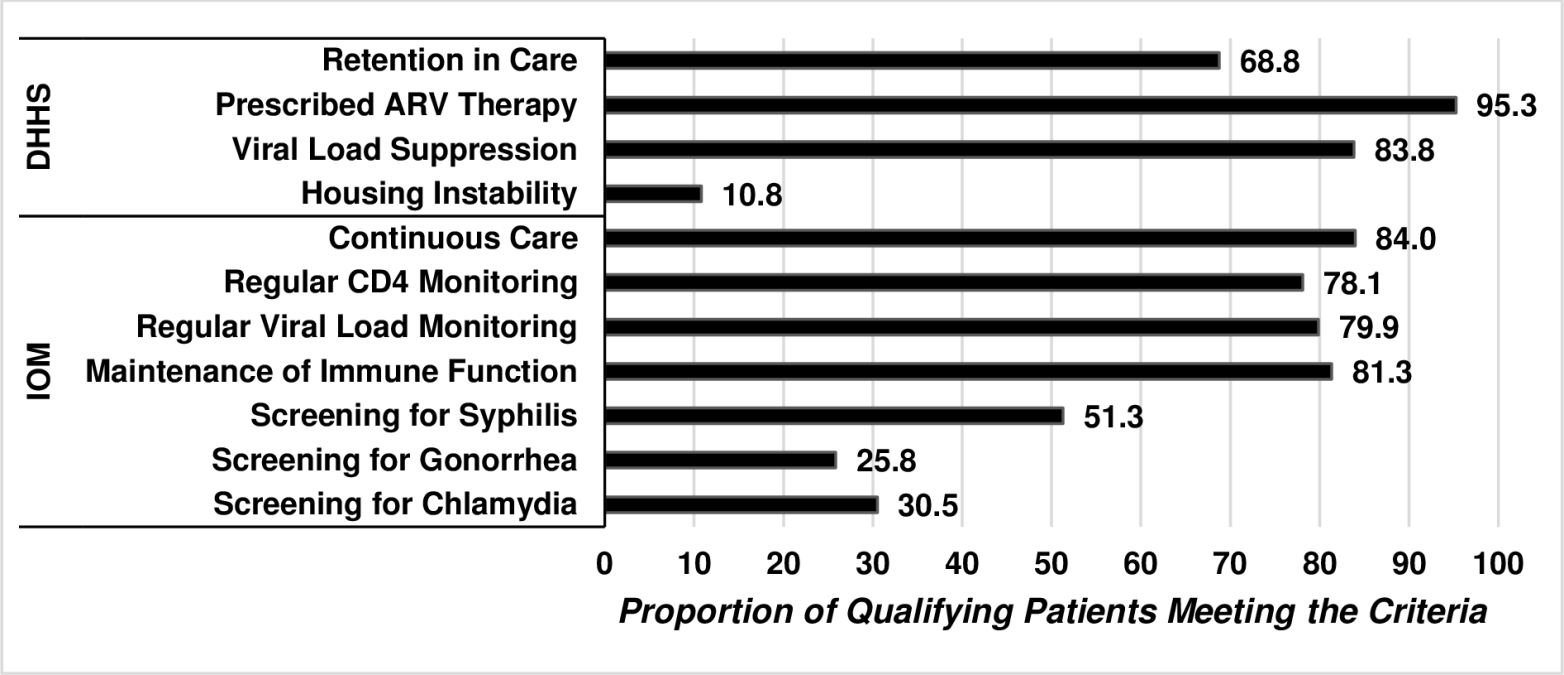
Proportion of DC cohort participants meeting criteria for selected HHS and IOM indicators for quality of care, DC cohort, 2011–2016. This figure represents individuals who were enrolled in the DC Cohort as of September 30, 2016 and met the criteria for selected Department of Health and Human Services and Institute of Medicine HIV quality of care indicators. While high proportions of participants met the HIV-related indicators (69%-95%), screening for sexually transmitted infections was relatively low (26%-51%).

For HHS-defined indicators, 69% of participants were retained in care, 95% had been prescribed ART, and 84% were virally suppressed at the end of their first year of follow-up (Fig 1). Although at enrollment, 8% of participants were homeless or temporarily housed, during the follow up period, 11% of participants were unstably housed. For IOM-defined indicators, 84% were in continuous care. The proportion of participants who received regular CD4 and VL monitoring was 78% and 80%, respectively. Among those in continuous care, 81% achieved and maintained immune function as measured by CD4 counts ≥ 350 cells/μl. The proportion of participants screened for syphilis, chlamydia, and gonorrhea was 51%, 31%, and 26%, respectively.

### Comparison of indicators by participant demographics

Differences in crude proportions for HHS-defined indicators were observed by demographic characteristics (Table 3). The proportion of participants retained in care was significantly higher among older participants (≥47 versus <47), injection drug users and those with ‘other’ risks for HIV and those who enrolled at community-based DC Cohort sites (versus hospital-based sites). After adjustment for sex, race/ethnicity, HIV risk group and clinic type, older participants were 1.5 times more likely to be retained in care compared to younger participants (OR: 1.5; 95% CI: 1.3, 1.8); those with ‘other’ HIV risk compared to MSM (OR: 2.8, 95%CI 1.8, 4.3); and those enrolled in community-based clinics were also more likely to be retained (OR: 1.7; 95% CI: 1.4, 2.0). The proportion of participants prescribed ARVs was significantly higher among older participants and males (p-value<0.0083 for both); no differences were observed by race, risk, or clinic type. After adjustment, older participants were more likely to be prescribed ARVs in multivariable analyses (OR: 1.5; 95% CI: 1.1, 2.1). The proportion of participants who were virally suppressed was significantly higher among older participants, males, whites, and MSM. After adjustment, older participants were nearly two times more likely to achieve viral suppression than their younger counterparts (OR: 1.8; 95% CI: 1.5, 2.2). In contrast, non-Hispanic blacks were 0.4 times less likely to achieve viral suppression compared with non-Hispanic whites (OR: 0.4; 95% CI: 0.3, 0.5) and persons with ‘other’ transmission risk were 0.5 times less likely than MSM (0R: 0.5; 95% CI: 0.3, 0.7); Table 3).

**Table 3:** Factors associated with select HHS-defined care indicators, DC Cohort, 2011–2016^a^^,^^b^^,^^c^.

	Retention in care				Prescribed ARV therapy				Viral load suppression			
	N	Row %	P-value	aOR (95% CI)	N	Row %	P-value	aOR (95% CI)	N	Row%	P-value	aOR (95% CI)
**Total**	3,450	68.8			6,795	95.3			5,806	83.8		
**Age**												
<47	1,556	64.6	**<0.0001**	Ref	3,342	94.5	**0.0013**	Ref	2,740	80.0	** <0.0001**	Ref
≥47	1,894	72.6		**1.5 (1.3, 1.8)**	3,453	96.1		**1.5 (1.1, 2.1)**	3,066	87.7	** **	**1.8 (1.5, 2.2)**
**Sex at birth**												
Male	2,510	69.0	0.4885	Ref	4,960	95.8	**0.0013**	Ref	4,282	85.4	** <0.0001**	Ref
Female	940	68.0		0.9 (0.7, 1.2)	1,835	94.0		0.7 (0.4, 1.0)	1,524	79.8	** **	0.9 (0.7, 1.1)
**Race**												
Black	2,754	69.1	0.4198	1.0 (0.8, 1.3)	5,298	95.2	0.5918	1.0 (0.6, 1.5)	4,420	81.6	** <0.0001**	**0.4 (0.3, 0.5)**
White	481	68.1		Ref	989	95.8		Ref	921	93.0	** **	Ref
Other/ Unknown^d^	215	65.8		0.9 (0.6, 1.4)	508	94.8		0.9 (0.4, 1.7)	465	89.4	** **	0.7 (0.4, 1.2)
**HIV Risk**^e^												
MSM	1,290	67.5	**<0.0001**	Ref	2,641	95.4	0.0793	Ref	2,310	86.6	** <0.0001**	Ref
HRH	1,083	66.8		0.9 (0.7, 1.2)	2,197	94.9		1.1 (0.7, 1.7)	1,862	82.2		0.9 (0.7, 1.1)
IDU	297	76.0		1.3 (0.9,1.8)	461	93.5		0.6 (0.3, 1.1)	392	83.2		0.7 (0.5, 1.1)
Other	224	80.3		**2.8 (1.8, 4.3)**	337	94.9		1.2 (0.6, 2.6)	237	68.5		**0.5 (0.3, 0.7)**
Unknown	556	68.3		0.9 (0.7, 1.2)	1,159	96.5		1.4 (0.9, 2.4)	1,005	85.6		1.0 (0.7, 1.3)
**Clinic Type**												
Hospital-based	1,495	63.8	** <0.0001**	Ref	3,190	95.3	0.8139	Ref	2,717	82.8	0.0203	Ref
Community-based	1,955	73.1		**1.7 (1.4, 2.0**)	3,605	95.2		1.0 (0.7, 1.3)	3,089	84.8		1.2 (1.0, 1.4)

^a^Row percent represents the proportion of participants who met the specific measure out of the population in each demographic group.

^b^P-value represents statistical differences by demographic characteristics; P-values less than 0.0083 are considered statistically significant, using a Bonferroni correction for multiple testing.

^c^Adjusted odds ratio (aOR) are adjusted for all other variables presented in the table; aORs in bold denote statistical differences at the 0.0083 level, using a Bonferroni correction for multiple testing.

^d^Other race includes races reported as Hispanic, Asian, American Indian/Native American, Hawaiian/Other Pacific Islander, White-Black, and Other.

^e^MSM risk includes persons identified as having both MSM and injection drug use risk. Other risk includes perinatal transmission (65%), transfusion/coagulation disorder (19%), hemophiliacs and risk due to occupational exposure and other (16%).

Note. HHS = Health and Human Services; ARV = antiretroviral; OR = odds ratio; CI = confidence interval; MSM = men who have sex with men; HRH = high risk heterosexual risk; IDU = injection drug use; CBO = community-based organization

Differences in crude proportions for IOM-defined indicators were observed by demographic characteristics (Table 4). The proportion receiving regular CD4 monitoring was higher among older participants, females, Blacks and those with ‘other’ risk. After adjustment, persons identified as ‘other/unknown’ race were less likely and persons identified as ‘other’ risk were more likely to undergo regular CD4 monitoring (OR: 0.6; 95% CI: 0.5, 0.9 and OR: 2.2; 95% CI: 1.4, 3.4, respectively). Significant differences were observed in regular VL monitoring by sex, race, HIV risk, and clinic type. After adjustment, persons identified with ‘other’ risk and those enrolled in community-based clinics were more likely to achieve VL monitoring (OR: 2.1; 95% CI: 1.4, 3.4 and OR: 1.3; 95% CI: 1.1, 1.5, respectively). Differences in crude proportions for maintenance of immune function were observed by sex, race, and HIV risk. After adjustment, persons identified as ‘other/unknown’ race were 0.5 times less likely to achieve maintenance of immune function compared to whites (OR: 0.5; 95% CI: 0.4, 0.8).

**Table 4:** Factors associated with select IOM-defined care indicators, DC Cohort, 2011–2016^a^^,^^b^^,^^c^.

	Regular CD4 monitoring				Regular viral load monitoring				Maintenance of immune function			
	N	Row^a^ %	P-value^b^	aOR^c^ (95% CI)	N	Row^a^ %	P-value^b^	aOR^c^ (95% CI)	N	Row %^a^	P-value^b^	aOR^c^ (95% CI)
**Total**	5,744	78.1			5,873	79.9			5,024	81.3		
** Age**												
<47	2,799	76.7	**0.0048**	Ref	2,881	79.0	0.0633	Ref	2,460	82.0	0.2305	Ref
≥47	2,945	79.4		1.2 (1.0, 1.4)	2,992	80.7		1.1 (1.0, 1.3)	2,564	80.8		0.9 (0.8, 1.1)
**Sex at birth**												
Male	4,123	76.9	**<0.0001**	Ref	4,229	78.9	**0.0007**	Ref	3,577	80.4	**0.0021**	Ref
Female	1,621	81.3		1.1 (0.9, 1.4)	1,644	82.5		1.1 (0.9, 1.3)	1,447	83.8		1.3 (1.0, 1.7)
**Race**^**d**^												
Black	4,559	79.8	**<0.0001**	1.2 (0.9, 1.5)	4,603	80.5	**0.0032**	1.1 (0.9, 1.4)	3,942	81.5	**<0.0001**	0.8 (0.6, 1.0)
White	815	75.1		Ref	825	76.0		Ref	730	84.4		Ref
Other/ Unknown	370	66.8		**0.6 (0.5, 0.9)**	445	80.3		1.2 (0.9, 1.7)	352	74.6		**0.5 (0.4, 0.8)**
**HIV Risk**^**e**^												
MSM	2,165	75.1	**<0.0001**	Ref	2,224	77.2	**<0.0001**	Ref	1,901	81.8	**0.0037**	Ref
HRH	1,898	80.2		1.1 (0.9, 1.4)	1,946	82.2		1.2 (1.0, 1.6)	1,700	82.9		1.0 (0.8, 1.3)
IDU	408	81.3		1.2 (0.8, 1.7)	414	82.5		1.3 (0.9, 1.8)	363	80.3		0.9 (0.6, 1.3)
Other	316	87.1		**2.2 (1.4, 3.4)**	316	87.1		**2.1 (1.3, 3.3)**	265	82.3		0.9 (0.6, 1.4)
Unknown	957	77.1		1.0 (0.8, 1.2)	973	78.4		1.0 (0.8, 1.2)	795	77.3		0.7 (0.6, 0.9)
**Clinic type**												
Hospital-based	2,682	76.9	0.0224	Ref	2,717	77.9	**0.0001**	Ref	2,260	81.6	0.6057	Ref
Community-based	3,062	79.1		1.2 (1.0, 1.4)	3,156	81.6		**1.3 (1.1, 1.5)**	2,764	81.1		1.0 (0.8, 1.2)

^a^Row percent represents the proportion of participants who met the specific measure out of the population in each demographic group.

^b^P-value correspond to bivariate comparison of characteristics; P-values less than 0.0083 are considered statistically significant, using a Bonferroni correction for multiple testing.

^c^Adjusted odds ratio (aOR) are adjusted for all other variables presented in the table; aORs in bold denote statistical differences at the 0.0083 level, using a Bonferroni correction for multiple testing.

^d^Other race includes races reported as Hispanic, Asian, American Indian/Native American, Hawaiian/Other Pacific Islander, White-Black, and Other.

^e^MSM risk includes persons identified as having both MSM and injection drug use risk. Other risk includes perinatal transmission (65%), transfusion/coagulation disorder (19%), hemophiliacs and risk due to occupational exposure and other (16%).

Note. IOM = Institute of Medicine; ARV = antiretroviral; OR = odds ratio; CI = confidence interval; MSM = men who have sex with men; HRH = high risk heterosexual risk; IDU = injection drug use; CBO = community-based organization

## Discussion

Using aggregate data from a clinic-attending population of PLWHA, we were able to create a comprehensive snapshot of a standard set of quality of care and HIV care continuum outcomes among an urban population of PLWHA to identify areas of success, gaps, and disparities in care outcomes. When comparing our HHS-defined retention-in-care estimate to other published estimates, we found that retention among DC Cohort participants was slightly lower than participants in NA-ACCORD (71–75%) but higher than that reported by the HIV Research Network and others (59%-65%) [4, 14–21]. The DC Cohort IOM continuity of care estimate was relatively high at 84% and higher than local surveillance estimates of 69%; however, this measure only relies on 12 months of follow-up [22]. Thus the IOM continuity of care measure may be a better indicator for initial or short term engagement in care, whereas the HHS indicator (which requires 24 months of observation) may be more useful for assessing longer-term follow-up.

The HHS-defined prescribed ARV estimate of 95% was higher than both the NA-ACCORD estimate of 82% and Veterans Aging Cohort Study (VACS) estimate of 91% [4, 23]. Similarly, our HHS-defined viral suppression estimate of 84% exceeded the DC surveillance estimates (57%), the Center for Disease Control’s National HIV Surveillance System estimate (48%), NA-ACCORD estimates (54–78%) and other large cohort estimates (66–76%) [5, 6, 16, 19, 24–26]. Further analysis to examine the role of specific ART regimens and viral suppression may be warranted to better characterize effective regimens among this population. With respect to routine immunologic and virologic monitoring, our estimates for regular CD4 and VL monitoring of 78% and 80%, respectively, were similar to the VACS estimate of 80% [23, 24]. These relatively high estimates indicate that patients enrolled in the DC Cohort are well engaged in HIV care.

In contrast, our estimates for routine screening for STIs were much lower compared to our higher rates of care continuity, ARV prescription, and viral suppression. The DC Cohort estimate for routine screening of gonorrhea (NG) and chlamydia (CT) were lower than combined testing estimates of NG/CT observed in other HIV clinical cohorts (26% and 31% versus 39–42% in other cohorts) [27, 28]. Similarly, the DC Cohort screening estimate for syphilis was lower than other HIV cohorts of persons in care (51% versus 65%) [28]. Given the recent increases in syphilis, gonorrhea, and chlamydia rates observed among PLWHA, particularly MSM, providers need to be reminded of the importance of secondary prevention and routine sexually transmitted disease screening among PLWHA [27–31]. Further analyses by demographic and transmission risk characteristics are needed to identify sub-groups least likely to be screened.

The disparities in care outcomes observed with regard to age, race, and housing have previously been documented in this Cohort and in other HIV cohort analyses; with older persons, whites, and those stably housed doing better than comparison populations [5, 32–36]. Similarly, the observation that perinatally infected participants, who comprise 65% of ‘other’ HIV risk, had a higher likelihood of being retained but lower likelihood of achieving viral suppression is consistent with previous Cohort findings and national trends [7, 32, 37]. Interestingly, we also observed differences by clinic type with participants at community-based clinics having more regular VL monitoring and better retention in care than those enrolled at hospital-based clinics. This observation differs from that of other studies that did not find meaningful differences in retention with respect to clinic type [38, 39]. Further exploration of patient characteristics by site, as well as characterization of care delivery such as the receipt of primary care and availability of ancillary services at each site, as well as the costs of providing these services, is necessary to fully understand the implications of these findings.

Measurement of these HHS and IOM quality of care estimates provides a baseline for the DC Cohort population and a representative snapshot of care delivery in the DC area. Continued monitoring of these outcomes in the future will allow us to compare groups and track changes over time. Monitoring of care patterns and clinical outcomes also allows for identification of disparities in the delivery and achievement of optimal care and opportunities to develop targeted programmatic interventions. Currently, all participating DC Cohort sites receive semi-annual benchmark reports which include aggregate site-specific data compared to overall Cohort data as well as lists of out-of-care patients, and patients with detectable viral loads who may need clinical intervention [10]. While this type of feedback is useful, we are currently developing more real-time clinical dashboards with select key indicators that providers can access and review, to allow for better monitoring, screening, and more timely intervention [40]. Moving forward, by collaborating with the DC DOH and the DC Cohort clinic sites, interventions may include strengthening patient navigation programs to flag patients in need of screening, who are at risk of falling out of care, or require other targeted intervention [41].

Our analysis has several limitations worth noting. Our results are only applicable to those PLWHA who were already linked to care and consented to be in the DC Cohort, thus we are unable to characterize the first few steps (i.e., HIV testing, linkage to care) of the care continuum. Additionally, our cohort generally represents those in established care; DC Cohort participants included in this analysis tended to have relatively high CD4 counts at enrollment (52%) and almost 60% had never had an AIDS diagnosis. Therefore, this limits our ability to estimate differences among those who may not be engaged in care and our findings may not be generalizable to other populations of PLWHA. Moreover, the quality of data depends on how well data are manually and electronically abstracted through disparate medical record systems; however, as part of routine DC Cohort data processes, clinical validations and data queries and audits are conducted regularly.

Despite these limitations, our initial assessments of the quality of care being provided in a large urban area with a high HIV prevalence provide evidence that care delivery among PLWHA at these participating clinics may be approaching national targets but that disparities remain at both the individual and clinic level [18]. By using these indicators as baseline measures of quality of care, we were able to identify gaps in care and, through our unique partnership with the DC DOH and the major HIV providers in the city, we intend to use this evidence-base to develop strategic and practical public health programs and interventions to optimize care delivery in Washington, DC.
